# Whole-body vibration ameliorates glial pathological changes in the hippocampus of hAPP transgenic mice, but does not affect plaque load

**DOI:** 10.1186/s12993-023-00208-9

**Published:** 2023-03-20

**Authors:** Tamas Oroszi, Eva Geerts, Reuben Rajadhyaksha, Csaba Nyakas, Marieke J. G. van Heuvelen, Eddy A. van der Zee

**Affiliations:** 1 Department of Neurobiology, Groningen Institute for Evolutionary Life Sciences (GELIFES), University of Groningen, Nijenborgh 7, 9747 AG Groningen, The Netherlands; 2 Research Center for Molecular Exercise Science, Hungarian University of Sports Science, Budapest, Hungary; 3 Department of Morphology and Physiology, Health Science Faculty, Semmelweis Univesity, Budapest, Hungary; 4 Department of Human Movement Sciences, University of Groningen, University Medical Center Groningen, Groningen, The Netherlands

**Keywords:** Passive exercise, Neuroinflammation, Amyloid beta, J20 mice, Motor coordination

## Abstract

**Background:**

Alzheimer’s disease (AD) is the core cause of dementia in elderly populations. One of the main hallmarks of AD is extracellular amyloid beta (Aβ) accumulation (APP-pathology) associated with glial-mediated neuroinflammation. Whole-Body Vibration (WBV) is a passive form of exercise, but its effects on AD pathology are still unknown.

**Methods:**

Five months old male J20 mice (n = 26) and their wild type (WT) littermates (n = 24) were used to investigate the effect of WBV on amyloid pathology and the healthy brain. Both J20 and WT mice underwent WBV on a vibration platform or pseudo vibration treatment. The vibration intervention consisted of 2 WBV sessions of 10 min per day, five days per week for five consecutive weeks. After five weeks of WBV, the balance beam test was used to assess motor performance. Brain tissue was collected to quantify Aβ deposition and immunomarkers of astrocytes and microglia.

**Results:**

J20 mice have a limited number of plaques at this relatively young age. Amyloid plaque load was not affected by WBV. Microglia activation based on IBA1-immunostaining was significantly increased in the J20 animals compared to the WT littermates, whereas CD68 expression was not significantly altered. WBV treatment was effective to ameliorate microglia activation based on morphology in both J20 and WT animals in the Dentate Gyrus, but not so in the other subregions. Furthermore, GFAP expression based on coverage was reduced in J20 pseudo-treated mice compared to the WT littermates and it was significantly reserved in the J20 WBV vs. pseudo-treated animals. Further, only for the WT animals a tendency of improved motor performance was observed in the WBV group compared to the pseudo vibration group.

**Conclusion:**

In accordance with the literature, we detected an early plaque load, reduced GFAP expression and increased microglia activity in J20 mice at the age of ~ 6 months. Our findings indicate that WBV has beneficial effects on the early progression of brain pathology. WBV restored, above all, the morphology of GFAP positive astrocytes to the WT level that could be considered the non-pathological and hence “healthy” level. Next experiments need to be performed to determine whether WBV is also affective in J20 mice of older age or other AD mouse models.

**Supplementary Information:**

The online version contains supplementary material available at 10.1186/s12993-023-00208-9.

## Introduction

Alzheimer’s disease (AD) has been identified as the most prevalent type of dementia [1]. The major pathological hallmarks of AD are extracellular amorphous amyloid plaques aggregated of misfolded amyloid beta peptides and intracellular neurofibrillary tangles consisting of hyperphosphorylated TAU proteins [1, 2]. However, there is growing evidence that the involvement of glial-mediated neuroinflammation may also play an important role in the pathogenesis of AD [3–8].

Given the high complexity and serious consequences of AD, there has been a broad range of scientific inquiries for pharmacological, as well as non-pharmacological treatment strategies [9, 10]. Ample meta-analytical reviews have summarized the effects of regular physical activity (PA) on the symptoms and progression of AD [11–13]. Although it is important to note that these studies often demonstrated inconsistent results and do not show unanimity, a growing body of evidence support the potential efficiency and value of regular and moderated PA to prevent and/or slow the progression of AD. Further, regular PA promotes neurogenesis, synaptic plasticity and angiogenesis via increases in the level of neurotransmitters and neurotrophic factors [12, 13]. The findings of these studies indicate that amyloid plaque formation and AD-related neuroinflammation could be reduced by these factors. Active exercise may therefore be a viable intervention to trigger anti-inflammatory effects to ameliorate amyloid plaque formation. Pharmacological and non-pharmacological AD treatments recently focus more on the function of astrocytes, although their role entails a complex balance between neurotoxic and neuroprotective effects depending on the disease stage and microenvironmental factors (Rodríguez-Giraldo et al. and references therein [14]). Moreover, there is a growing need for alternative exercise strategies to support populations who are unable and/or unmotivated to perform sufficient PA due to their limited cognitive and/or motor capabilities.

Whole body vibration (WBV), a form of passive exercise using mechanical vibration platforms, may provide an alternative for PA. Benefits of WBV in older populations are reflected by improved general fitness, mobility and balance [15, 16]. In rodents, WBV is able to increase neuromuscular dynamics and muscle strength [17], to improve adipose tissue dysfunction and glucose metabolism [18], and to promote muscle healing [19]. Recent animal studies have shown that vibration stimulates hippocampal functioning reflected in improved modulation of synaptic and/or neural plasticity and spatial memory, and in alleviated pathological changes of glial cells [20–25].

To the best of our knowledge, and confirmed by a recent review [26], the therapeutic effects of WBV have never been investigated in the context of AD pathophysiology. However, similar alternative therapies based on transcranial ultrasound and auditory stimulation proved the therapeutic efficacy of mechanical waves to mitigate AD pathophysiology. Given the potential significance of WBV for neural protection, we hypothesized that WBV may beneficially modulate the glial activation (known to be critically involved in AD pathology; see Rodríguez-Giraldo et al., 2022 for review [14]) and amyloid beta plaque formation in the hippocampus of transgenic human APP-J20 mice, a well-known model of AD [27]. To achieve this aim, we evaluated a five-week long WBV protocol in five months-old transgenic human APP-J20 mice with the main focus on AD molecular pathophysiology. Further, since WBV has been widely acknowledged to improve muscle parameters, minor attention was also paid to the evaluation of motor performance. The age of five months was chosen to start the experiment because at this age J20 mice show an early, but detectable age-related deficit in cognitive and behavioral performance [28–31], as well as in the progression of neuroinflammation and plaque formation [32].

## Materials and methods

### Animals

Twenty-six transgenic hAPP-J20 male mice (PDGFB-APPSwInd; C57Bl6/J background) and 24 male wild type (WT) littermates (C57Bl6/J) serving as healthy controls were used in this experiment. The age of these animals was 5 months at the start of the experiment. Both J20 and WT mice were randomly allocated to a WBV group [WBV—J20 (n = 13) and WBV – WT (n = 12)] or a control group [pseudo WBV—J20 (n = 13) and pseudo WT (n = 12)]. Pseudo control mice were subjected to the same environmental stimuli, including placement on the vibration plate and sound of the vibration plate, but were not exposed to vibration. Animals were individually housed during the intervention period. Individual housing started one week before the start of the intervention. Food and water were available ad libitum. Animals were housed under standard laboratory conditions (12/12 dark—light cycle (lights on at 9:00 a.m.), temperature (22 ± 1 °C) and humidity control (50 ± 10%)). Health status of the animals was checked daily and their body weight was registered each week by the researchers. All experimental procedures were evaluated and approved by the national Central Authority for Scientific Procedures on Animals (CCD) and by the local Institutional Animal Welfare Body of University of Groningen (IvD).

### Whole-body vibration procedure

**Fig. 1:**
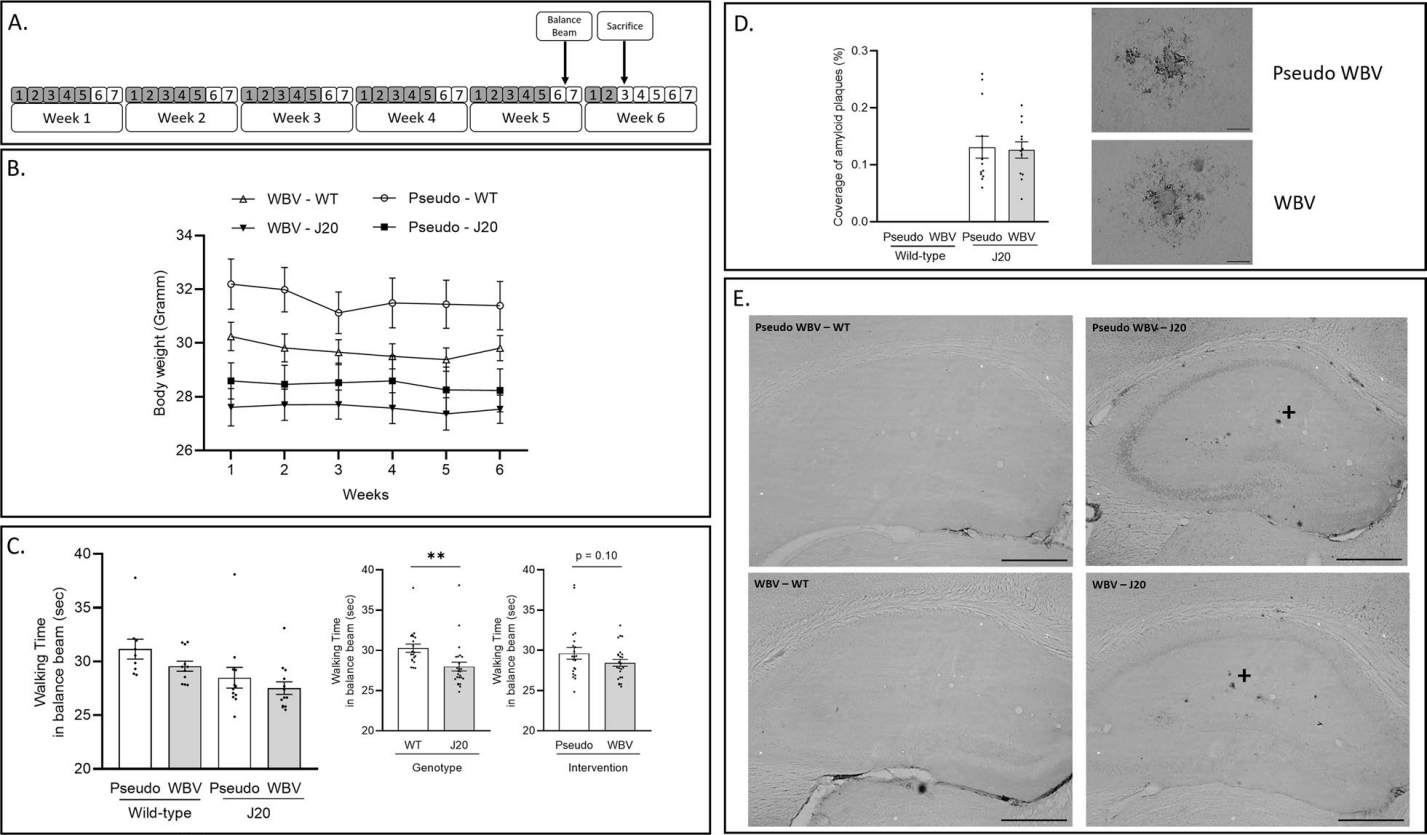
Experimental design (**A**): 5 months old male hAPP-J20 mice and their wild (WT) littermates underwent 5 weeks of whole body vibration intervention (WBV) with twice daily session of 10 min exposure, five times per week (gray color marks the days of treatment). After 5 weeks, balance beam test was performed to assess motor coordination. Animals were terminated on week 6 at the age of ~ 6.5 months and brain tissue was collected for immunohistological analyses. Effects of intervention (pseudo vs. WBV) and genotype (J20 vs. WT) on body weight (**B**), motor performance (**C**) and plaque load (**D**). WT animals showed significatly higher body weight during the intervention compared to the J20 animals (**B**). Walking distance in the balance beam test was only significantly improved in the J20 animals compared to the WT (**C**). Amyloid plaque deposition in the hippocampus was not significantly affected by WBV intervention (**D**). Images of 6e10 were taken about the whole hippocampus at 50 × magnifications to visualze total amyloid plaque distribution (**E**), representative images of areas marked by + are depicted in **D**. Data are depicted as mean ± SEM. ** indicates: P < .01. Scale bars in **D** are 50 um and in **E** are 500 um

We adhered to the new reporting guidelines for WBV studies in animals [33]. Animals were exposed to a vibration session of 10 min twice per day (i.e.: at 10 a.m. and 16 p.m.), five times per week during 5 consecutive weeks (Fig. 1A). Furthermore, animals also received two WBV days on week 6, but only one session on the second day (i.e.: WBV session at 10 a.m.; 24 h before sacrifice). The WBV device has been described before [34, 35]. In short, this device consists of an oscillator (LEVELL R.C. Oscillator Type TG200DMP), a power amplifier (V406 Shaker Power Amplifier) and a cage (44.5 × 28 × 16 cm) separated by 12 removable compartments (6.5 × 7.5 × 20) attached to the oscillator. Throughout a WBV/pseudo WBV session, mice were randomly placed into these individual compartments and were exposed to constant vertical vibrations with a frequency of 30 Hz, an amplitude of 50 micron (100 micron peak-to-peak displacement) and of a sinusoidal nature. These parameters of vibration were verified by additional measurements using a 3D-accelerometer [34]. The individual compartments and the cage were cleaned with 70% ethanol and dry paper tissue between each training session.

Animals did not receive prior habituation to the experimental settings. Both J20 and WT mice showed slightly exited behavior during the first week of intervention. This general unprompted activity was reduced from the second week onwards. Some escape attempts (i.e.: jumping) were recognized in the J20 mice during the entire intervention. Similarly, a significantly higher degree of defecation (i.e.: number of pellets) was observed in both J20 and WT mice after the WBV sessions in the first week of intervention, which seemed to be normalized only in the WT mice from the second week onwards. In contrast, J20 mice showed this higher degree of defecation after each training session throughout the entire intervention. All vibration sessions were performed in the housing room of the animals. Finally, the animals did not show any kind of acute, short-term and/or long-term side effects by vibration.

### Balance beam

The balance beam test was conducted after 5 weeks of WBV treatment to assess sensorimotor coordination with main focus on the functionality of hind limbs [34]. A 1 m long wooden beam (with diameter of 4.5 mm) was placed horizontally 60 cm above the floor. The home cage of the tested animal was positioned at the end of the beam, serving as motivation and target factor for the animal.

Mice were familiarized to the experimental setup by two progressive trials (placed on the beam 10 cm and 40 cm away from the target) and subsequently performed four test trials (at 100 cm distance), with 30 s break between all trials to ensure enough recovery to avoid potential injuries induced by muscle fatigue. Video records were taken during the procedures, which were independently analyzed by two researchers. Time needed to cross the beam served as measure of performance and the mean of the three best trials was used as final outcome variable. If an animal was unable or unwilling to cross over the beam it was excluded from the final statistical analysis (1 animal from the pseudo WBV/J20 group; 1 animal from the WBV/WT group; 3 animals from the pseudo WBV/WT group). The beam was cleaned by 70% ethanol and dry paper tissue after each animal.

### Immunohistochemistry

Mice were anesthetized with pentobarbital and transracially perfused with saline and 4% paraformaldehyde (PFA) twenty-four hours after the last WBV session. Brain tissue was harvested and postfixed by 4% PFA for 24 h before being transferred to phosphate buffer (PB) for 3 days. After 3 days of washing, brains were dehydrated by 30% sucrose solution and frozen by liquid nitrogen. Brains were stored at − 80 °C until coronal sectioning (20 μm) on a cryostat. Immunohistochemistry was performed to determine microglia and astrocyte features, as well as plaque deposition in the dorsal hippocampus. Free floating sections were used for all staining procedures.

*Microglia detection.* Ionized calcium binding adaptor molecule 1 (IBA1) staining was performed to visualize the morphological state of microglia cells; and cluster of differentiation factor 68 (CD68) was done to determine the level of microglia activation biochemically. Sections were incubated for 3 days at 4 °C by the following primary antibodies: 1) Rabbit anti IBA1 (Wako, SKN4887, 1:2500) in 0.01 M phosphate buffer saline (PBS) containing 1% bovine serum albumin (BSA) and 0.1% Triton-X (TX) or 2) Rat anti CD68 (BioRad, MCA1957GA; 1:1000) in 0.01 M tris buffered saline (TBS) with 5% BSA, 5% normal donkey serum (NDS).

*Astrocyte detection.* Glial fibrillary acidic protein (GFAP) immunohistochemistry was performed to detect astrocyte volume. Sections were pre—incubated in TBS containing 3% BSA and 0.1% TX followed by incubation of primary antibody (Cell Signaling, E4L7M, 1:10000).

*Plaque detection.* Beta amyloid 1–16 (6E10) was stained to detect plaque deposition in the hippocampus. Sections were pre-incubated in 0.01 M TBS with 0.1% TX and 3% normal goat serum before the overnight incubation of primary antibody (BioLegend, SIG-39320; 1:2000).

In addition, prior to all stainings, endogenous peroxidase activity was blocked by hydrogen peroxidase (H_2_O_2_) (0.3% for IBA1, GFAP and 6e10; 1% for CD68). For detection, we used biotinylated anti-mouse secondary antibodies (IBA1 and GFAP: Goat Anti Rabbit 1: 500; CD68: Mouse Anti Rat 1:500; 6E10: Goat Anti Mouse 1:400) followed by processing with ABC kit (Vectastain ABC kit, Vector Laboratories) and developed our signal with diaminobenzidine (Sigma Fast, Cat: D4418) and 0.1% H2O2. All sections were intensively washed during the staining processes in PBS or TBS. Sections were mounted on gelation-coated slides, and placed overnight in a drying cabinet. Finally, sections were dehydrated in graded solutions of ethanol and xylol and cover slipped.

### Microscopy

Number of microglia, cell body size, dendrites size and total coverage were determined in the Cornu Ammonis 1 (CA1), Cornu Ammonis 3 (CA3), Dentate Gyrus (DG) and Hilus regions of the hippocampus based on the IBA1 staining (200 × magnification). Since microglia activation based on morphology is defined as shortened dendritic processes and increased cell body size, the ratio of the cell body to total cell size was calculated (see [36] for details) as the outcome measure of microglia activation.

The coverage (% of the area of interest covered by the immunostaining) of CD68 and GFAP positive cells were determined in the CA1, CA3, DG and Hilus regions (20 × magnification). Similarly, coverage of 6E10 was measured in the dorsal hippocampus (40 × magnification). All analyses were performed by Image J software.

### Statistical analysis

Statistical analysis was performed by Statistica 13.2 software. 2 × 2 factorial ANOVAs were performed with intervention (vibration/pseudo vibration) and genotype (J20/WT) as factors and, in case of significant intervention x genotype interaction, followed by Tukey’s post hoc test to reveal statistical differences between the four groups in balance beam performance, IBA1, GFAP and CD68 stainings. In addition, amyloid plaque deposition between the two J20 groups was compared using independent T-tests. Mixed design repeated measurements ANOVA was performed with intervention (WBV, pseudo WBV) and genotype (J20, WT) as between-subjects factors and time course (week 1–5) as within-subjects factor to analyze differences in body weight. Statistical significance was set at p < 0.05. Graphs were created by GraphPad Prism 8 software. Descriptive data were expressed as mean ± SEM.

## Results

### Body weight and motor performance

Mixed design repeated measures ANOVA showed a significantly higher body weight in the WT groups compared to the J20 animals (main effect genotype: F_(1.46)_ = 15.060; p < 0.001) (Fig. 1B). In addition, a strong tendency of lower body weight was observed in the WBV treated groups compared to the pseudo-WBV groups (main effect intervention: F_(1.46)_ = 3.978; p = 0.052). A significant effect of time course was also revealed (main effect time course: F_(5.230)_ = 4.871; p < 0.001). Further, the interaction of genotype*time course showed significant difference (intervention genotype x time interaction: F_(5.230)_ = 2.788; p = 0.018) and additional post-hoc analysis revealed decreased body weight in the WT animals for week 1 vs. week 3–6 (post hoc p < 0.05), but not in the J20s.

The balance beam test was used in week 5 to assess motor coordination and functionality of primarily the hind limbs. Two-way ANOVA revealed that the J20 animals showed significantly better walking performance compared to the WT animals (Main effect genotype: F_(1.41)_ = 9.410; p = 0.003) (Fig. 1C). In addition, a trend of improved motor coordination was found in the vibration treated groups compared with pseudo vibration treated animals (vibration treated animals showed decreased walking time on the beam), but it did not reach statistical significance (Fig. 1C). No significant interaction effect of intervention and genotype was observed in the balance beam test.

### Plaque formation

To determine whether WBV affects the level of amyloid deposition in J20 mouse, the total coverage of 6e10 staining was measured in the hippocampus (Hilus, DG, CA3, and CA1 subregions pooled). An early plaque load was found in the hippocampus of J20 mice at the age of six months. In contrast, plaques were not present in the hippocampus of their WT littermates. Furthermore, plaque load was not significantly different between WBV and pseudo-WBV treated J20 groups (Fig. 1D). Representative images of amyloid plaque load are depicted in Fig. 1E.

### Microglia

To determine whether differences in microglia activation in the subregions of hippocampus (CA1, CA2, DG and Hilus) existed across genotype and WBV intervention, activated microglia were identified by the expression of IBA1 and CD68 positive cells; two frequently used markers for microglia.

**Fig. 2:**
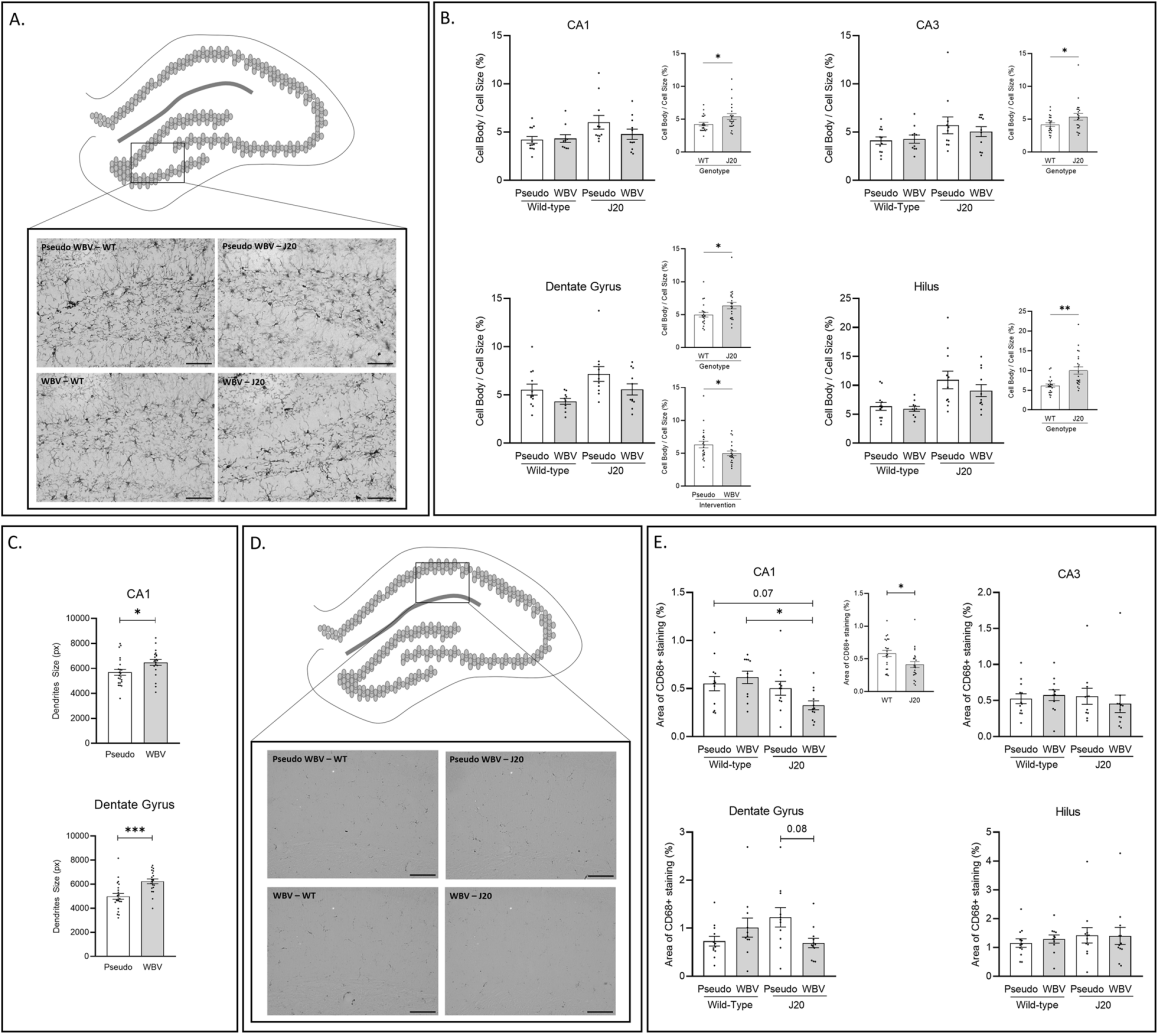
Microglia visualized by IBA1 staining in the Hilus subregions is depicted in (**A**). Effects of intervention (pseudo vs. WBV) and genotype (WT vs. J20) on microglia activation in the CA1, CA3, DG and Hilus subregions are depicted in (**B**). Significant increase of microglia activation was observed in the J20 animals compared to the WT controls in all subregions (**B**, CA1, CA3, Dentate Gyrus and Hilus). Microglia activation was only significantly decreased by WBV in the DG (**B**, Dentate Gyrus). Furthermore, WBV treatment significantly increased the size of microglia dendritic processes in the CA1 and DG subregions (**C**, CA1 and Dentate Gyrus). Expression of CD68 coverage in the CA1 subregion is visualized in** D**. Significant decrease of CD68 expression in the CA1 area was detected in the J20 animals compared to the WT controls (**E**, CA1). In addition, a tendency (p = 0.06) of interaction effect (intervention vs. genotype) on CD68 expression was also observed in the CA1 subregion; additional post-hoc analysis showed a significant decrease for the J20—WBV group compared to the WT—WBV group, as well as the same tendency (p = 0.07) compared to the WT—pseudo WBV group (**E**, CA1). A significant effect of interaction (intervention vs. genotype) in CD68 expression was observed in the DG area; additional post hoc revealed a strong tendency (p = 0.08) of decrease CD68 expression in the J20—WBV treated animals compared to the J20—pseudo WBV controls (**E**, Dentate Gyrus). CD68 expression was not significantly altered in the CA3 and Hilus areas (**E**, CA3 and Hilus). Representative Images of IBA1 and CD68 were taken about the Hilus and CA1 subregions at 200 × magnifications to visualze microglia (**A** and **C**). Relevant statistical differences are marked in **B**, **C** and **D**. Data are depicted as mean ± SEM. * indicates: P = .05. Scale bars in **A** and **D** are 100 um

Morphological parameters of microglia based on the IBA1 immunostaining were determined including number, total coverage, cell body and dendrites area in the CA1, CA3, DG and Hilus hippocampal subregions. Representative images of IBA1 expression in the Hilus subregion are visualized in Fig. 2A. Significantly decreased total coverage and increased cell body size were detected in the J20 animals compared to the WT controls in all hippocampal subregions. It was also observed that WBV significantly ameliorated the size of dendritic processes in the CA1 (Main effect intervention: F_(1.40)_ = 5.636; p = 0.022) and DG subregions (Main effect intervention: F_(1.40)_ = 15.15; p < 0.001) (Fig. 2C). Microglia number was not significantly altered. These morphological outcomes are summarized in Table 1. Furthermore, microglia activation was calculated based on the IBA1 expression as the ratio of the cell body to total cell size. Microglia activation of the 4 investigated subregions are summarized in Fig. 2B. Significantly higher degree of microglia activation was detected in the J20 groups compared to their WT littermates in the CA1, CA3, DG and Hilus subregions (Fig. 2B). In addition, decreased microglia activation in the DG subregion was observed in the WBV vs. pseudo-WBV groups (Main effect intervention: F_(1.40)_ = 5.738; p = 0.021) (Fig. 2B).

**Table 1:** Effects of intervention (pseudo WBV vs. WBV) and genotype (J20 vs. WT) on microglia number (n), microglia coverage (in %), cell body size and dendrites size measured in pixels, and the number of cells in the CA1, CA3, DG and Hilus subregions of the hippocampus

Group	Region	Microglia number (n)	Total coverage (in %)	Cell body area (px)	Dendrites area (px)
Pseudo WBV—WT	CA1	33 ± 1.9	16 ± 0.9	237 ± 15	5647 ± 342
	CA3	33 ± 2.0	16 ± 1.1	244 ± 12	6094 ± 531
	DG	31 ± 1.9	16 ± 0.9	270 ± 16	4993 ± 393
	Hilus	25 ± 2.0	15 ± 1.1	262 ± 21	4201 ± 344
Pseudo WBV—J20	CA1	33 ± 1.1	13 ± 0.6 +	353 ± 39 +	5728 ± 332
	CA3	32 ± 2.0	13 ± 0.5 +	328 ± 42 +	5734 ± 510
	DG	33 ± 1.0	12 ± 0.4 +	365 ± 41 +	4978 ± 277
	Hilus	21 ± 1.5	11 ± 0.6 +	398 ± 41 +	3464 ± 214
WBV—WT	CA1	36 ± 2.7	16 ± 0.8	257 ± 17	5952 ± 404*
	CA3	32 ± 1.8	16 ± 0.7	252 ± 14	5948 ± 379
	DG	31 ± 1.9	16 ± 0.7	267 ± 14	6129 ± 261*
	Hilus	21 ± 2.0	14 ± 0.8	271 ± 12	4460 ± 308
WBV—J20	CA1	34 ± 1.9	15 ± 0.7 +	348 ± 45 +	6950 ± 163*
	CA3	31 ± 1.6	15 ± 0.9 +	349 ± 35 +	6764 ± 355
	DG	34 ± 1.6	14 ± 0.7 +	368 ± 43** + **	6335 ± 299*
	Hilus	22 ± 2.7	11 ± 0.7 +	361 ± 42 +	3829 ± 448

Microglia number was not significantly altered by genotype or intervention. Significantly higher coverage was detected in the J20 mice compared to the WT mice in all subregions. In contrast, cell body size was significantly increased in the J20 animals compared to the WT animals. WBV treatment significantly increased the size of dendritic processes in both the J20 and WT mice in the CA1 and DG areas. Data are depicted as mean ± SEM. + indicates a significant difference between the main factors “wild type vs. J20”

^*^indicates a significant difference between main factors “WBV vs. pseudo WBV”. Additional figures related to these parameters can be found in the Additional file 1

Total coverage of CD68 staining, a protein highly expressed by activated microglia, was measured in the same subregions of hippocampus. Representative images of CD68 in the CA1 subregion are visualized in Fig. 2D. Data of the 4 investigated subregions are depicted in Fig. 2E. CD68 coverage in the CA1 region was significantly decreased by the J20 genotype (main effect genotype: F_(1.43)_ = 6.767; p = 0.012). In addition, a tendency of intervention x genotype interaction was also observed (p = 0.06) with a lower coverage in the CA1 region for J20-WBV vs. WT pseudo-WBV (post-hoc: 0.07) and WT-WBV animals (post-hoc: 0.01). Furthermore, a significant intervention x genotype effect was found in the DG subregions (F_(1.42)_ = 6.999; p = 0.011). Further post-hoc analysis showed a tendency of lower CD68 coverage in the J20-WBV group compared to the J20 pseudo-WBV controls (post-hoc p = 0.08).

### Astrocytes

**Fig. 3:**
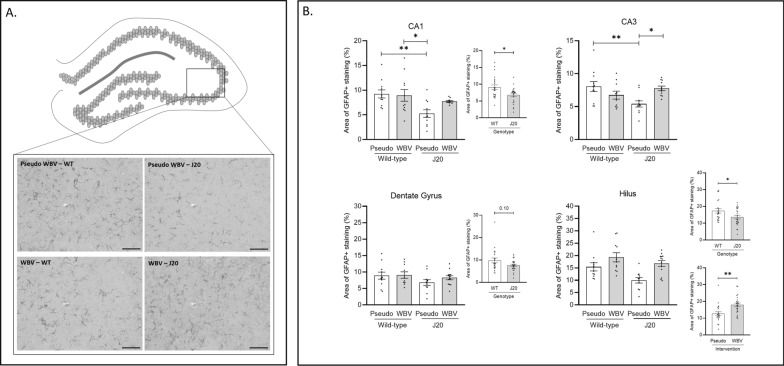
GFAP + astrocytes in the CA3 hipocampal subregion are visualized in (**A**). Effects of intervention (pseudo vs. WBV) and genotype (J20 vs. WT) on expression of GFAP positive cells in the CA1, CA3, DG and Hilus regions are depicted in **B**. Significantly decreased coverage of GFAP positive cells in the J20 animals was detected in the CA1 and Hilus regions (**B**, CA1 and Hilus). The same trend was observed in the CA3 and Hilus subregions (**B**, CA3 and Hilus). Significant effect of interaction (intervention vs. genotype) was detected in the CA1 and CA3 subregions (**B**, CA1 and CA3). Additional posthoc analysis revealed that WT—WBV and pseudo WBV groups showed significantly higher coverage compared to the J20 – pseudo WBV group in the CA1 region (**B**, CA1). Significantly increased GFAP coverage was detected in the J20—WBV and WT—pseudo WBV groups compared to the J20—pseudo WBV group in the CA3 region (**B**, CA3). In addition, significant increase of GFAP coverage was detected by WBV in the Hilus region (Panel **B**, Hilus). Representative images of GFAP + astrocytes were taken about the CA3 hippocampal subregion at 200 × magnifications (**A**). Data are depicted as mean ± SEM. * indicates: P = .05. ** P < .01. *** P < .001. Scale bars in A are 100 um

To determine whether hippocampal astrocyte volume would be affected by WBV and/or genotype, covered area of GFAP positive cells were quantified in CA1, CA3, DG and Hilus hippocampal subregions. Representative images of GFAP in the CA3 subregion are visualized in Fig. 3A. Data of the 4 investigated subregions are also summarized in Fig. 3B. Two-way factorial ANOVA revealed a significantly lower volume of GFAP positive cells in the CA1 and Hilus subregions of J20 animals compared to their WT littermates (Fig. 3B. Further, WBV significantly increased astrocyte coverage in the Hilus subregions (F_(1.41)_ = 20.96; p < 0.001) (Fig. 3B. A genotype x intervention was found in the CA3 subregion (F_(1.39)_ = 10.58; p = 0.002). It was found that WT animals showed lower coverage in WBV vs. pseudo group, whereas the J20 animals had higher coverage in WBV vs. pseudo group. Additional post-hoc analysis revealed that the coverage in CA3 region was significantly higher for WBV vs. pseudo-WBV in J20 mice (post hoc = 0.023) and it was lower for J20 – pseudo-WBV vs. WT pseudo-WBV mice (post-hoc = 0.009) (Fig. 3B). Consistent with these observations, a similar trend was detected in the CA1 region, but this interaction effect did not reach statistical significance.

## Discussion

The aim of this experiment was to investigate the therapeutic impact of long-term (five weeks) WBV intervention with low intensity of a sinusoidal nature on amyloid deposition, neuroinflammation and motor performance during the early stage of AD in hAPP-J20 transgenic mice. Our results demonstrated that exposure of J20 mice to WBV for five weeks ameliorated the early progression of astroglial pathology. In contrast, WBV did not influence microglia activation or the amyloid plaque load in the human APP-J20 mouse model.

A reduction in volume (coverage) of GFAP positive astrocytes in the hippocampus was found in J20 vs. WT mice including the CA1, DG and Hilus. This is in line with other reports showing a reduction in volume of GFAP positive astrocytes in J20 mice of 5–6 months of age, however, it was not visible at 8 months indicating that astrocyte population may only decrease during the early stage of AD pathogenesis [37, 38]. In contrast to these results, a significantly increased coverage of GFAP positive astrocytes has been reported in J20 mice at 6 and 9 months of age [32, 39]. These previously reported findings suggest the presence of an early AD-related deficit; however, certain recovery mechanisms may become activated later on in life. These findings are most likely due to the complex balance between neurotoxic and neuroprotective effects depending on the disease stage and microenvironmental factors (Rodríguez-Giraldo et al., 2022 and references therein [14]). Although WBV did ameliorate the astroglial pathological changes in J20 mice, it did not reduce plaque load. This is in line with the findings from Wang et al. that early activation of astrocytes at the age of 3–5 months does not influence the deposition of amyloid plaques in the brains of J20 mice [40].

As far as we know, neither WBV as a form of passive exercise nor active exercise have been investigated regarding hippocampal functioning in the human APP-J20 mouse model. However, another form of cognitive stimulation, known as enrichment environment, has been reported to prevent astroglial volume and morphological changes in the early stage of AD in the hippocampus of J20 mice [37]. Long-term environmental enrichment restored the astrocyte parameters similar to the age-matched non-transgenic control animals. In their design, no exercise devices (for instance: running wheel or disc) were included to ensure mainly cognitive stimulation by the enrichment environment. In our current study, we demonstrated similar effects that altered astrocyte morphology regarding AD progression can be reversed by exposure to a long-term WBV intervention. Hence, WBV seems to be able to mimic the effects of enrichment environment in the early stage of AD in J20 animals.

One of the mostly emphasized effects of WBV is stimulating and improving the musculoskeletal system. This is based on ample clinical and pre-clinical studies [41–43]. In rodents, low-intensity WBV improves neuromuscular dynamics, muscle strength and motor coordination [17, 22, 23, 34]. Although this neuromuscular response to WBV appears to be a pivotal adaptation, only a trend of improved motor coordination was found in the present study for both J20 and WT mice. J20 animals outperformed the WT littermates in the balance beam test. This is most likely due to the known hyperactivity of the J20 mice. Altered locomotor activity such as hyperactivity and disturbed home cage activity has been commonly reported in AD mouse models including J20 mice, which are often associated with increased amyloid levels and disease progression [44]. The onset of these disturbances varies between different models, however, the J20 model is one of them that seems to develop disruptions the earliest (around 1 month of age) [44]. We hypothesize, that the J20 animals approached the upper limit of their performance due to their disturbed locomotor behavior and thereby contributed to the mitigation of WBV’s effects on motor coordination. In addition, this trend of improved motor performance appears to be more pronounced in the WT animals. This observation seems to be in line with our previous studies reported in young mice [34] and old rats [22, 23].

We found in earlier studies from our research group that long-term (5 weeks) WBV has broad effects in young mice including improvements in motor performance, memory functions and levels of neurotransmitters [34, 35, 45]. In contrast, WBV did not influence the body weight of the animals in these studies [34, 35, 45]. A possible explanation is that here we used WBV twice per day, instead of once. The extra physical activity that comes with the handling procedure (for both the WBV and the pseudo-WBV groups) could account for this finding. However, it should be noted that the observed reduction of body weight over the time course of the intervention is very small and probably biologically irrelevant for the mice.

In accordance with the literature, we found an early plaque load in the hippocampus of J20 mice at the age of 6.5 months. Further, the number and volume of amyloid beta plaques seem to be comparable to those that have been reported in 5–6 months old J20 mice [37, 39]. Although fewer animals with a relatively high plaque load were observed after WBV, no significant overall reduction was found. Apparently WBV did not affect plaque load, although studies using ultrasound-based vibrational alternatives seem to reduce plaque load in various AD models [46–50]. However, it is important to emphasize that these experiments do not show unanimity regarding outcome measures and methodical approaches, and direct comparison with WBV is limited. Our results may though suggest that the vibratory aspect of ultrasound-based therapies is not the main causal factor for the observed findings.

There is evidence for the toxicity of amyloid beta regarding glial activation including both microglia and astrocytes [6–8]. We found significantly increased microglia activation (based on morphology and predominantly an increase in cell body size) in all hippocampal subregions of J20 animals compared to their WT littermates. In contrast, CD68 positive cells only showed an increase in the CA1 region. These observed discrepancies between both microglia markers could be related to the complex early events in the progression of neuroinflammation. Available data from literature also indicate that a significantly increased number of activated CD68 and IBA1 positive microglia cells was detectable in the hippocampus of J20 mice at 6–9 months of age compared to age-matched WT controls [37, 39]. Our findings seem to be in line with these previously reported observations. It was also found that WBV was able to ameliorate microglia activation in both J20 and WT animals in the DG subregions. This finding seems to be associated with increased dendritic processes of microglia in the DG and CA1 regions. However, despite the same tendency microglia activation in the CA1 regions was not significantly altered by WBV treatment. Furthermore, WBV did not affect cell body size in all investigated subregions. This finding indicates that WBV might be a more beneficial stimulus in the DG to shift microglia towards a more ramified morphology due to amelioration of dendritic processes, such as sensing the surrounding tissue. These discrepancies in the observed parameters might also be related to the relative early stage of the disease. Taken together, the beneficial effects of WBV on hippocampal microglia activation have been reported recently. Our research group found that WBV is able to reduce aging-related neuroinflammation associated with higher degree of microglia ramification in 18 months old rats [23]. Our current findings seem to be in line with this observation. Furthermore, it was reported by others, that long-term WBV intervention alleviates increased level of microglia immunostaining and reversed the decreased level of GFAP positive astrocytes in the CA1 hippocampal subregions of Sprague–Dawley rats induced by restrain stress test [21]. These findings suggest that WBV intervention in older J20 mice may yield different results as seen here in young J20 mice due to increased responsiveness of the microglia.

The positive impact of WBV on the functioning of compromised astrocytes is a novel finding. Traditionally, astrocytic pathology is characterized by an increase in the volume of GFAP-positive astrocytes, most notably seen in astrogliosis (see, for review, Kim et al., 2018 and references therein [51]). However, a decrease in the volume of hippocampal GFAP-immunoreactive astrocytes has been found in relation to depression and mood-disorders in human tissue and after posttraumatic stress disorder in rats [52, 53]. A decrease in the volume or coverage is most likely caused by a retraction of the astrocytic processes which will cause a decreased participation of astrocytic end feet in the tripartite synapse [54]. Hence, it could be that this type of astrocytic pathology reduces the uptake of excess of synaptic glutamate, leading to increased risk of excitotoxicity as also suggested by others [53]. Reduced astrocytic functioning due to shrinkage also negatively affects the release of neurotrophic factors [51]. We therefore interpret the decrease of GFAP in our data as a sign of pathology, and the recovery by WBV to the levels as seen in control animals as the prevention of pathology or its reversal if it was already present before we started the WBV intervention.

Restoring the volume (coverage) of GFAP-positive astrocytes could promote cellular signaling and synaptic plasticity, functions know to be sensitive to WBV [20; 45]. Both astrocytes and microglia can modify their morphology in response to their direct cellular vinicity [6–8] and are endowed with receptors for different types of neurotrophic factors [55–57] and neurotransmitters [58]. For instance: astrocytes posess TrkB1 receptors, the binding site of the neurotrophic factor BDNF, as well as cholinergic, serotonergic and dopaminergic receptors. These findings suggest that these morphological alterations could be mediated through multiple pathways and might be crucial for neural activity, synaptic plasticity and maintenance [58, 59]. It is also known that WBV exposure can stimulate the release of various neurotransmitters in different brain regions including the hippocampus [45, 60, 61], potentially supporting neuronal activity and health. Similarly, an increased level of BDNF was also detected after long-term vibration interventions [21, 25]. Populations of reactive astrocytes localized around the senile plaques could be able to produce, together with microglia, a wide range of pro-inflammatory molecules and contribute to the inflammatory state [62]. WBV may have the therapeutic potential to mitigate the level of pro-inflammatory factors after brain damage [25]. This preventive effect of WBV on astroglia volume might also be associated with the mitigation of pro-inflammatory responses.

## Limitations

Some limitations need to be addressed. Although the design of our WBV protocol was chosen and planned carefully, it required the use of pseudo-control groups to determine the sole effect of the vibrations. The control animals underwent pseudo-treatment and may also experience improvements in reducing the progression of amyloid pathology, as a result of the exposure to a new environment (i.e. the compartments of the vibration plate).

A significant decline of body weight was detected during the first two weeks of the intervention in the WT animals, but not in the J20 animals (time course * genotype). Further, a strong tendency of lower body weight was explored in the WBV treated group (main factor: intervention), however, the interaction of time course and intervention was not significantly altered (time course * intervention). Although the animals underwent prior habituation to the experimental room and its conditions before the start of the intervention, this indicates that the WT animals might have experienced some discomfort or stress or have been more sensitive during the first two weeks of the intervention. Over the last years, we have not experienced this kind of fluctuation in body weight in mice or rats in our former experiments [22, 23, 34, 35, 45]. Also, neither WBV nor pseudo WBV did influence the body weight of mice in our previous works with the same WBV device and settings [34, 35, 45]. We made the same observations in rats [22, 23]. This minor decline in body weight only in WT mice indicates that modifications in habituation and handling procedures may be considered in future projects. Finally, the WT animals had significantly higher body weights compared to the J20 (main effect genotype). Whether this difference between WT and J20 animals in body weight influenced the efficiency of the WBV treatment is unknown.

### Recommandations for future research

The initially stated aim of this study was to determine the contextual underlying mechanisms that might contribute to the beneficial effects of WBV in the J20 mouse model, a well-known model for AD. While recognizing the limitations of this research, we think, that we were able, at least in part, to achieve our objectives. We have identified the involvement of astrocytes in WBV-mediated effects. These findings can lead to specific outcomes in determining the research questions and strategies of future studies. Based on the available body of literature regarding WBV and AD (as well the J20 mouse model), we concluded that future studies should examine the influence of WBV on cognition and behavior including depression, anxiety and memory functions, as well as on further molecular markers related to neurogenesis, synaptic plasticity, growth factors, inflammatory and other neural markers. All of these research objectives could be relevant future perspectives in relation to AD and WBV.

## Conclusion

The results of this study suggest that glial changes in the early phase of amyloid pathology could be prevented by chronic (five weeks) exposure to low-intensity WBV. Our results contribute to the understanding of glial plasticity in response to WBV, which can be considered a new, potential therapeutic approach for neurodegenerative diseases. The clinical relevance has yet to be determined, and may be restricted to the early phase of AD as we found that WBV in late phase AD patients could not improve their cognitive performance, despite the demonstrated feasibility of WBV for (fragile) AD patients [63]. Our results seem to be consistent with the existing literature and indicate that glial cells can respond to vibrational stimuli adopting their volume to the condition as was found in the control mice. The underlying mechanism(s) could implicate parallel molecular and cellular responses such as altered energy metabolism, recycling and/or release of neurotransmitters and neurotrophic factors, especially in the plastic brain areas like the hippocampus. Whether these mechanisms indeed play a key role has to be determined in future studies. Further, the understanding and unraveling of these underlying mechanisms by translational scientific investigations can contribute to more advanced and effective study procedures and WBV protocols as indicated earlier by our research group [64].

In conclusion, the possibility that the progression of derailing of microglial and astroglial activation such as seen in neuroinflammation in the early stage of AD (and most likely other types of neurodegenerative diseases) can be slowed by application of WBV, as a passive alternative for active exercise, puts forward WBV as a treatment strategy worthwhile to pursuit. Notably for those unable to participate in active exercise protocols.

## Supplementary Information

**Additional file 1: Figure S1.** Effects of genotype x time course (**panel A**) and time course (**panel B**) were observed on body weight. Body weight was significantly decreased from week 1 - 2 to week 3 – 6 in the wild type animals. In contrast, this effect was not observed in the J20 animals. Significant decrease of body weight (effect of tiem course) was also revealed on week 1 vs. week 3, 5 and 6.

## Data Availability

The dataset(s) supporting the conclusions of this article is(are) included within the article (and its Additional File 1)**.**
