# Supplementary material for: Whole-body vibration ameliorates glial pathological changes in the hippocampus of hAPP transgenic mice, but does not affect plaque load
Source: Behav Brain Funct. 2023 Mar 20;19:5. doi: 10.1186/s12993-023-00208-9 (PMC10026461; doi:10.1186/s12993-023-00208-9)
Supplement: Supplementary file 1 — Additional file 1: Figure S1. Effects of genotype x time course (panel A) and time course (panel B) were observed on body weight. Body weight was significantly decreased from week 1 - 2 to week 3 – 6 in the wild type animals. In contrast, this effect was not observed in the J20 animals. Significant decrease of body weight (effect of tiem course) was also revealed on week 1 vs. week 3, 5 and 6. [file 12993_2023_208_MOESM1_ESM.docx]

| **Descriptive statistics** | | | | | | | | |
| --- | --- | --- | --- | --- | --- | --- | --- | --- |
| **Variable** | **Groups** | **N** | **Mean** | **Median** | **Min** | **Max** | **Std.Dev** | **SEM** |
| **Body Weight**  **Area under Curve** | All | 50 | 89.63800 | 87.70000 | 76.60000 | 112.2000 | 8.071464 | 1.141477 |
|  | WBV – J20 | 13 | 84.47692 | 83.00000 | 76.60000 | 102.3000 | 6.307556 | 1.749401 |
|  | Pseudo – J20 | 13 | 87.03846 | 86.10000 | 77.60000 | 106.0000 | 7.104405 | 1.970407 |
|  | WBV – Wild Type | 12 | 91.05000 | 90.15000 | 82.80000 | 99.00000 | 4.994815 | 1.441879 |
|  | Pseudo – Wild Type | 12 | 96.63333 | 95.00000 | 86.10000 | 112.2000 | 8.471807 | 2.445600 |
| **Balance beam**  **Walking Time** | All | 45 | 29.00556 | 28.75000 | 24.85000 | 38.10000 | 2.783601 | 0.414955 |
|  | WBV – J20 | 13 | 27.52692 | 26.65000 | 25.50000 | 33.10000 | 2.120005 | 0.587984 |
|  | Pseudo – J20 | 12 | 28.49167 | 27.57500 | 24.85000 | 38.10000 | 3.361266 | 0.970314 |
|  | WBV – Wild Type | 11 | 29.55909 | 29.50000 | 27.80000 | 31.80000 | 1.548680 | 0.466945 |
|  | Pseudo – Wild Type | 9 | 31.15000 | 30.55000 | 28.75000 | 37.80000 | 2.792512 | 0.930837 |
| **6E10**  **Coverage** | All | 25 | 0.128699 | 0.117600 | 0.040000 | 0.260000 | 0.059399 | 0.011880 |
|  | WBV – J20 | 12 | 0.126108 | 0.126417 | 0.040000 | 0.204667 | 0.049250 | 0.014217 |
|  | Pseudo – J20 | 13 | 0.131090 | 0.101833 | 0.060125 | 0.260000 | 0.069428 | 0.019256 |
| **GFAP Coverage**  **CA1** | All | 45 | 7.794089 | 7.591000 | 1.669000 | 16.52700 | 3.026910 | 0.451225 |
|  | WBV – J20 | 12 | 7.746458 | 7.619250 | 6.918000 | 8.746000 | 0.497242 | 0.143541 |
|  | Pseudo – J20 | 11 | 5.253636 | 4.747000 | 1.669000 | 10.08400 | 2.545729 | 0.767566 |
|  | WBV – Wild Type | 11 | 8.937864 | 7.099500 | 3.739000 | 16.52700 | 3.934724 | 1.186364 |
|  | Pseudo – Wild Type | 11 | 9.242727 | 8.586000 | 6.266500 | 15.21650 | 2.688751 | 0.810689 |
| **GFAP Coverage**  **CA3** | All | 43 | 6.997256 | 7.061500 | 2.810500 | 13.59200 | 2.078276 | 0.316934 |
|  | WBV – J20 | 11 | 7.772727 | 7.607000 | 6.093000 | 9.431500 | 1.135698 | 0.342426 |
|  | Pseudo – J20 | 11 | 5.402773 | 5.182500 | 2.810500 | 8.230000 | 1.559752 | 0.470283 |
|  | WBV – Wild Type | 10 | 6.739150 | 6.475750 | 4.642500 | 10.07300 | 1.924424 | 0.608556 |
|  | Pseudo – Wild Type | 11 | 8.050909 | 8.036500 | 5.080000 | 13.59200 | 2.517766 | 0.759135 |
| **GFAP Coverage**  **Hilus** | All | 45 | 15.11029 | 14.40100 | 3.379000 | 29.11650 | 5.495450 | 0.819213 |
|  | WBV – J20 | 13 | 16.77312 | 18.63250 | 9.838000 | 22.24550 | 4.200068 | 1.164889 |
|  | Pseudo – J20 | 11 | 9.972955 | 10.69400 | 3.379000 | 16.80800 | 3.669952 | 1.106532 |
|  | WBV – Wild Type | 11 | 19.29405 | 17.37700 | 11.78700 | 29.11650 | 6.123266 | 1.846234 |
|  | Pseudo – Wild Type | 10 | 13.99755 | 13.15150 | 10.54700 | 19.11200 | 2.911651 | 0.920745 |
| **GFAP Coverage**  **DG** | All | 45 | 8.240073 | 7.797500 | 1.887000 | 15.60450 | 2.970313 | 0.442788 |
|  | WBV – J20 | 13 | 8.251600 | 8.712250 | 4.161500 | 12.50250 | 2.217707 | 0.615081 |
|  | Pseudo – J20 | 11 | 6.805682 | 6.923000 | 1.887000 | 11.77975 | 2.886018 | 0.870167 |
|  | WBV – Wild Type | 10 | 9.073350 | 7.795500 | 5.299250 | 13.90050 | 3.132231 | 0.990498 |
|  | Pseudo – Wild Type | 11 | 8.903318 | 8.333000 | 4.155000 | 15.60450 | 3.509666 | 1.058204 |
| **CD68 Coverage**  **CA1** | All | 47 | 0.496175 | 0.502300 | 0.099000 | 1.102000 | 0.241272 | 0.035193 |
|  | WBV – J20 | 12 | 0.325818 | 0.290250 | 0.121000 | 0.662500 | 0.160511 | 0.046336 |
|  | Pseudo – J20 | 12 | 0.502300 | 0.502300 | 0.099000 | 1.102000 | 0.247951 | 0.071577 |
|  | WBV – Wild Type | 11 | 0.616300 | 0.656000 | 0.259500 | 0.853500 | 0.214797 | 0.064764 |
|  | Pseudo – Wild Type | 12 | 0.550292 | 0.556750 | 0.246000 | 1.082500 | 0.254793 | 0.073552 |
| **CD68 Coverage**  **CA3** | All | 46 | 0.526779 | 0.454568 | 0.075000 | 1.715000 | 0.321398 | 0.047388 |
|  | WBV – J20 | 12 | 0.454136 | 0.400250 | 0.123000 | 1.715000 | 0.420551 | 0.121403 |
|  | Pseudo – J20 | 11 | 0.559333 | 0.551000 | 0.220500 | 1.539000 | 0.368883 | 0.111222 |
|  | WBV – Wild Type | 11 | 0.574185 | 0.582704 | 0.075000 | 1.025500 | 0.249663 | 0.075276 |
|  | Pseudo – Wild Type | 12 | 0.526125 | 0.463500 | 0.188500 | 1.023500 | 0.237234 | 0.068484 |
| **CD68 Coverage**  **Hilus** | All | 47 | 1.318430 | 1.242500 | 0.145000 | 4.270000 | 0.758337 | 0.110615 |
|  | WBV – J20 | 12 | 1.403182 | 1.113500 | 0.369500 | 4.270000 | 1.023727 | 0.295524 |
|  | Pseudo – J20 | 12 | 1.422350 | 1.362675 | 0.145000 | 3.981000 | 0.921313 | 0.265960 |
|  | WBV – Wild Type | 11 | 1.292350 | 1.353000 | 0.268500 | 2.122500 | 0.470200 | 0.141771 |
|  | Pseudo – Wild Type | 12 | 1.153667 | 1.103000 | 0.497000 | 2.329500 | 0.507074 | 0.146380 |
| **CD68 Coverage**  **DG** | All | 46 | 0.906220 | 0.772750 | 0.104250 | 2.690750 | 0.551632 | 0.081334 |
|  | WBV – J20 | 12 | 0.691523 | 0.654125 | 0.304500 | 1.513500 | 0.338054 | 0.097588 |
|  | Pseudo – J20 | 11 | 1.226425 | 1.175000 | 0.157000 | 2.688750 | 0.672615 | 0.202801 |
|  | WBV – Wild Type | 11 | 1.012675 | 0.833500 | 0.104250 | 2.690750 | 0.662674 | 0.199804 |
|  | Pseudo – Wild Type | 12 | 0.729813 | 0.645750 | 0.222250 | 1.535750 | 0.349256 | 0.100822 |
| **IBA1**  **Microglia Activation**  **CA1** | All | 44 | 4.823918 | 4.278121 | 2.416108 | 11.11822 | 1.797282 | 0.270950 |
|  | WBV – J20 | 11 | 4.777370 | 3.944299 | 2.759640 | 8.216708 | 1.750496 | 0.527794 |
|  | Pseudo – J20 | 11 | 6.008796 | 4.734706 | 4.040796 | 11.11822 | 2.335524 | 0.704187 |
|  | WBV – Wild Type | 10 | 4.328376 | 4.093668 | 3.302262 | 7.212023 | 1.260611 | 0.398640 |
|  | Pseudo – Wild Type | 12 | 4.193401 | 3.691913 | 2.416108 | 6.437741 | 1.232694 | 0.355848 |
| **IBA1**  **Microglia Activation**  **CA3** | All | 44 | 4.778098 | 4.595804 | 2.196341 | 13.27111 | 1.967905 | 0.296673 |
|  | WBV – J20 | 11 | 5.040157 | 5.217603 | 2.579890 | 6.789118 | 1.698750 | 0.512193 |
|  | Pseudo – J20 | 11 | 5.698001 | 5.130218 | 3.036535 | 13.27111 | 2.903536 | 0.875449 |
|  | WBV – Wild Type | 10 | 4.271909 | 4.125971 | 2.454318 | 6.887127 | 1.338420 | 0.423246 |
|  | Pseudo – Wild Type | 12 | 4.116458 | 4.125914 | 2.196341 | 6.361359 | 1.326634 | 0.382966 |
| **IBA1**  **Microglia Activation**  **DG** | All | 44 | 5.681360 | 5.062430 | 2.678514 | 13.73177 | 2.124133 | 0.320225 |
|  | WBV – J20 | 11 | 5.572675 | 4.520382 | 2.987387 | 8.402872 | 1.904576 | 0.574251 |
|  | Pseudo – J20 | 11 | 7.165920 | 6.677606 | 4.258100 | 13.73177 | 2.532865 | 0.763688 |
|  | WBV – Wild Type | 10 | 4.327801 | 4.506606 | 2.678514 | 5.553887 | 0.953751 | 0.301603 |
|  | Pseudo – Wild Type | 12 | 5.548107 | 4.876027 | 2.890406 | 9.988471 | 1.965411 | 0.567365 |
| **IBA1**  **Microglia Activation**  **Hilus** | All | 44 | 8.084048 | 6.996407 | 3.242414 | 21.69973 | 3.825777 | 0.576758 |
|  | WBV – J20 | 11 | 9.078767 | 7.498307 | 4.902077 | 14.95052 | 3.438113 | 1.036630 |
|  | Pseudo – J20 | 11 | 10.92244 | 9.657419 | 5.477401 | 21.69973 | 5.026621 | 1.515583 |
|  | WBV – Wild Type | 10 | 5.936823 | 5.907005 | 3.734025 | 8.369823 | 1.375855 | 0.435083 |
|  | Pseudo – Wild Type | 12 | 6.359713 | 6.291049 | 3.242414 | 10.62619 | 2.338784 | 0.675149 |
| **IBA1**  **Cell Body**  **CA1** | All | 44 | 299.0574 | 277.5935 | 155.3276 | 666.5198 | 115.9316 | 17.47734 |
|  | WBV – J20 | 11 | 348.9388 | 297.4534 | 193.3286 | 660.8350 | 149.8493 | 45.18125 |
|  | Pseudo – J20 | 11 | 353.5304 | 307.2043 | 264.6574 | 666.5198 | 130.4205 | 39.32326 |
|  | WBV – Wild Type | 10 | 257.9255 | 283.4343 | 155.3276 | 335.1742 | 56.85129 | 17.97796 |
|  | Pseudo – Wild Type | 12 | 237.6757 | 252.8374 | 157.9486 | 314.0415 | 53.98049 | 15.58283 |
| **IBA1**  **Cell Body**  **CA3** | All | 44 | 293.5497 | 267.4051 | 174.7844 | 712.6494 | 104.7152 | 15.78642 |
|  | WBV – J20 | 11 | 349.5107 | 349.5660 | 183.1346 | 526.1742 | 116.1716 | 35.02706 |
|  | Pseudo – J20 | 11 | 328.8600 | 294.1200 | 205.9781 | 712.6494 | 141.7203 | 42.73027 |
|  | WBV – Wild Type | 10 | 252.3109 | 261.0677 | 196.9063 | 338.0260 | 46.24155 | 14.62286 |
|  | Pseudo – Wild Type | 12 | 244.2500 | 257.6156 | 174.7844 | 307.3916 | 44.84607 | 12.94595 |
| **IBA1**  **Cell Body**  **DG** | All | 44 | 318.0365 | 293.1385 | 167.9365 | 734.7143 | 113.5127 | 17.11268 |
|  | WBV – J20 | 11 | 368.2257 | 293.2782 | 222.9399 | 639.1947 | 143.7515 | 43.34270 |
|  | Pseudo – J20 | 11 | 365.3625 | 325.7512 | 261.8453 | 734.7143 | 136.9271 | 41.28507 |
|  | WBV – Wild Type | 10 | 267.8702 | 282.8495 | 200.0103 | 319.4426 | 46.87520 | 14.82324 |
|  | Pseudo – Wild Type | 12 | 270.4528 | 267.0473 | 167.9365 | 364.0116 | 56.78246 | 16.39169 |
| **IBA1**  **Cell Body**  **Hilus** | All | 44 | 323.3106 | 291.2689 | 175.0323 | 717.8750 | 119.6168 | 18.03291 |
|  | WBV – J20 | 11 | 361.2520 | 325.2358 | 178.9694 | 577.1818 | 140.7255 | 42.43033 |
|  | Pseudo – J20 | 11 | 398.4274 | 349.5632 | 227.3411 | 717.8750 | 138.9493 | 41.89478 |
|  | WBV – Wild Type | 10 | 271.7126 | 266.9028 | 222.4571 | 345.3481 | 40.67452 | 12.86241 |
|  | Pseudo – Wild Type | 12 | 262.6722 | 258.5749 | 175.0323 | 448.2159 | 74.22675 | 21.42742 |
| **IBA1**  **Dendrites**  **CA1** | All | 44 | 6062.720 | 6071.222 | 3592.446 | 8442.125 | 1155.717 | 174.2309 |
|  | WBV – J20 | 11 | 6950.426 | 7013.113 | 6196.004 | 8039.547 | 541.3064 | 163.2100 |
|  | Pseudo – J20 | 11 | 5728.310 | 5625.098 | 3592.446 | 7987.130 | 1104.410 | 332.9921 |
|  | WBV – Wild Type | 10 | 5952.655 | 6123.279 | 4093.830 | 8442.125 | 1280.613 | 404.9655 |
|  | Pseudo – Wild Type | 12 | 5647.254 | 5054.460 | 4606.949 | 7838.536 | 1185.379 | 342.1895 |
| **IBA1**  **Dendrites**  **CA3** | All | 44 | 6138.831 | 5779.320 | 3248.871 | 11407.49 | 1519.613 | 229.0903 |
|  | WBV – J20 | 11 | 6764.242 | 6851.072 | 5070.565 | 9189.710 | 1179.417 | 355.6075 |
|  | Pseudo – J20 | 11 | 5734.977 | 5465.128 | 3248.871 | 9391.920 | 1693.751 | 510.6852 |
|  | WBV – Wild Type | 10 | 5948.787 | 5698.594 | 4617.759 | 8800.571 | 1199.548 | 379.3305 |
|  | Pseudo – Wild Type | 12 | 6094.108 | 5617.719 | 4092.750 | 11407.49 | 1840.614 | 531.3394 |
| **IBA1**  **Dendrites**  **DG** | All | 44 | 5583.373 | 5689.667 | 3200.421 | 8161.723 | 1203.795 | 181.4790 |
|  | WBV – J20 | 11 | 6335.730 | 6327.967 | 3985.380 | 7566.527 | 991.9743 | 299.0915 |
|  | Pseudo – J20 | 11 | 4978.397 | 4667.295 | 3703.224 | 6587.137 | 920.2149 | 277.4552 |
|  | WBV – Wild Type | 10 | 6129.080 | 6220.357 | 4786.235 | 7410.738 | 826.3586 | 261.3175 |
|  | Pseudo – Wild Type | 12 | 4993.517 | 4876.498 | 3200.421 | 8161.723 | 1363.580 | 393.6317 |
| **IBA1**  **Dendrites**  **Hilus** | All | 44 | 3982.906 | 3898.548 | 2328.087 | 8041.100 | 1156.243 | 174.3102 |
|  | WBV – J20 | 11 | 3829.941 | 3555.334 | 2459.922 | 8041.100 | 1486.826 | 448.2949 |
|  | Pseudo – J20 | 11 | 3464.051 | 3918.284 | 2586.138 | 4366.033 | 709.8661 | 214.0327 |
|  | WBV – Wild Type | 10 | 4460.066 | 4295.843 | 3150.057 | 6199.702 | 975.8978 | 308.6060 |
|  | Pseudo – Wild Type | 12 | 4201.109 | 4280.538 | 2328.087 | 6155.650 | 1194.789 | 344.9059 |
| **IBA1**  **Coverage**  **CA1** | All | 44 | 15.19682 | 15.02750 | 10.17000 | 21.11000 | 2.890460 | 0.435753 |
|  | WBV – J20 | 11 | 15.06045 | 15.13500 | 10.32000 | 18.40500 | 2.360398 | 0.711687 |
|  | Pseudo – J20 | 11 | 13.21136 | 13.19000 | 10.17000 | 16.88000 | 2.048218 | 0.617561 |
|  | WBV – Wild Type | 10 | 16.14450 | 15.50250 | 11.32000 | 20.25500 | 2.636079 | 0.833601 |
|  | Pseudo – Wild Type | 12 | 16.35208 | 16.90000 | 11.99500 | 21.11000 | 3.444153 | 0.994241 |
| **IBA1**  **Coverage**  **CA3** | All | 44 | 15.89597 | 15.61750 | 10.68000 | 24.45000 | 3.131024 | 0.472020 |
|  | WBV – J20 | 11 | 15.92182 | 15.75500 | 11.08500 | 20.85000 | 3.168982 | 0.955484 |
|  | Pseudo – J20 | 11 | 13.94682 | 14.27500 | 10.68000 | 16.59000 | 1.876646 | 0.565830 |
|  | WBV – Wild Type | 10 | 16.72500 | 16.75500 | 12.39500 | 20.42000 | 2.305545 | 0.729077 |
|  | Pseudo – Wild Type | 12 | 16.96813 | 18.03500 | 12.39000 | 24.45000 | 3.995389 | 1.153370 |
| **IBA1**  **Coverage**  **DG** | All | 44 | 14.97753 | 14.51875 | 9.177500 | 21.37000 | 2.779692 | 0.419054 |
|  | WBV – J20 | 11 | 14.77148 | 14.69500 | 9.177500 | 17.92250 | 2.408274 | 0.726122 |
|  | Pseudo – J20 | 11 | 12.95227 | 13.31750 | 11.06500 | 14.82250 | 1.484616 | 0.447629 |
|  | WBV – Wild Type | 10 | 16.01850 | 15.87625 | 12.13250 | 20.06250 | 2.448163 | 0.774177 |
|  | Pseudo – Wild Type | 12 | 16.15542 | 17.17750 | 11.63250 | 21.37000 | 3.373653 | 0.973890 |
| **IBA1**  **Coverage**  **Hilus** | All | 44 | 13.32386 | 12.93750 | 7.015000 | 21.90500 | 3.410342 | 0.514128 |
|  | WBV – J20 | 11 | 11.95318 | 12.32000 | 7.465000 | 15.28500 | 2.597803 | 0.783267 |
|  | Pseudo – J20 | 11 | 11.33000 | 11.61000 | 7.015000 | 15.01000 | 2.287456 | 0.689694 |
|  | WBV – Wild Type | 10 | 14.69400 | 14.37750 | 9.735000 | 18.16000 | 2.722512 | 0.860934 |
|  | Pseudo – Wild Type | 12 | 15.26625 | 15.37250 | 10.03000 | 21.90500 | 4.102611 | 1.184322 |
| **IBA1**  **Cell Size**  **CA1** | All | 44 | 6322.081 | 6218.106 | 3971.525 | 8731.458 | 1185.892 | 178.7799 |
|  | WBV – J20 | 11 | 7140.579 | 7181.075 | 5522.947 | 8572.203 | 819.5926 | 247.1165 |
|  | Pseudo – J20 | 11 | 6081.841 | 5944.251 | 3971.525 | 8310.561 | 1113.865 | 335.8430 |
|  | WBV – Wild Type | 10 | 6210.581 | 6433.047 | 4399.071 | 8731.458 | 1303.849 | 412.3133 |
|  | Pseudo – Wild Type | 12 | 5884.930 | 5246.481 | 4764.897 | 8141.496 | 1191.880 | 344.0661 |
| **IBA1**  **Cell Size**  **CA3** | All | 44 | 6456.199 | 6018.633 | 4368.750 | 11663.67 | 1478.210 | 222.8485 |
|  | WBV – J20 | 11 | 7113.753 | 7111.577 | 5424.828 | 9457.732 | 1178.751 | 355.4067 |
|  | Pseudo – J20 | 11 | 6159.111 | 5699.923 | 4660.878 | 9686.040 | 1549.800 | 467.2824 |
|  | WBV – Wild Type | 10 | 6201.098 | 5938.521 | 4955.785 | 9022.000 | 1178.458 | 372.6613 |
|  | Pseudo – Wild Type | 12 | 6338.358 | 5903.036 | 4368.750 | 11663.67 | 1834.092 | 529.4567 |
| **IBA1**  **Cell Size**  **DG** | All | 44 | 6112.135 | 6077.806 | 4028.975 | 8679.108 | 1132.644 | 170.7525 |
|  | WBV – J20 | 11 | 6703.956 | 6616.158 | 4351.113 | 8205.722 | 1043.199 | 314.5364 |
|  | Pseudo – J20 | 11 | 5323.758 | 5095.242 | 4028.975 | 6871.271 | 940.5557 | 283.5882 |
|  | WBV – Wild Type | 10 | 6396.950 | 6532.188 | 5058.936 | 7611.792 | 821.7718 | 259.8670 |
|  | Pseudo – Wild Type | 12 | 6054.966 | 5683.117 | 4896.205 | 8679.108 | 1272.683 | 367.3920 |
| **IBA1**  **Cell Size**  **Hilus** | All | 44 | 4267.490 | 4221.805 | 2604.779 | 8455.600 | 1147.099 | 172.9317 |
|  | WBV – J20 | 11 | 4191.193 | 3843.534 | 2891.933 | 8455.600 | 1530.683 | 461.5182 |
|  | Pseudo – J20 | 11 | 3862.479 | 4169.314 | 3048.897 | 4658.725 | 620.8760 | 187.2012 |
|  | WBV – Wild Type | 10 | 4731.779 | 4608.152 | 3372.514 | 6497.036 | 977.2854 | 309.0448 |
|  | Pseudo – Wild Type | 12 | 4321.784 | 4139.824 | 2604.779 | 7166.571 | 1229.967 | 355.0610 |
| **IBA1**  **Microglia Number**  **CA1** | All | 44 | 34.35227 | 33.75000 | 22.50000 | 58.00000 | 6.423593 | 0.968393 |
|  | WBV – J20 | 11 | 34.22727 | 35.00000 | 22.50000 | 47.50000 | 6.345722 | 1.913307 |
|  | Pseudo – J20 | 11 | 33.31818 | 32.50000 | 26.00000 | 40.00000 | 3.938735 | 1.187573 |
|  | WBV – Wild Type | 10 | 36.25000 | 35.25000 | 24.00000 | 58.00000 | 8.740868 | 2.764105 |
|  | Pseudo – Wild Type | 12 | 33.83333 | 31.00000 | 25.00000 | 45.50000 | 6.589707 | 1.902285 |
| **IBA1**  **Microglia Number**  **CA3** | All | 44 | 32.47727 | 31.00000 | 21.00000 | 47.00000 | 6.183205 | 0.932153 |
|  | WBV – J20 | 11 | 31.50000 | 33.50000 | 22.50000 | 39.00000 | 5.329165 | 1.606804 |
|  | Pseudo – J20 | 11 | 32.31818 | 30.50000 | 24.00000 | 45.50000 | 6.961583 | 2.098996 |
|  | WBV – Wild Type | 10 | 33.35000 | 34.25000 | 21.00000 | 43.50000 | 5.995600 | 1.895975 |
|  | Pseudo – Wild Type | 12 | 32.79167 | 30.75000 | 24.00000 | 47.00000 | 6.955895 | 2.007994 |
| **IBA1**  **Microglia Number**  **DG** | All | 44 | 32.64205 | 32.50000 | 21.75000 | 43.75000 | 5.492965 | 0.828096 |
|  | WBV – J20 | 11 | 34.20455 | 34.00000 | 22.50000 | 41.50000 | 5.423120 | 1.635132 |
|  | Pseudo – J20 | 11 | 33.31818 | 32.50000 | 29.75000 | 39.25000 | 3.437097 | 1.036324 |
|  | WBV – Wild Type | 10 | 31.87500 | 31.37500 | 24.50000 | 43.50000 | 6.164696 | 1.949448 |
|  | Pseudo – Wild Type | 12 | 31.22917 | 31.75000 | 21.75000 | 43.75000 | 6.615631 | 1.909768 |
| **IBA1**  **Microglia Number**  **Hilus** | All | 44 | 22.84091 | 21.00000 | 10.00000 | 43.00000 | 6.980682 | 1.052377 |
|  | WBV – J20 | 11 | 22.90909 | 20.50000 | 10.00000 | 38.00000 | 9.082451 | 2.738462 |
|  | Pseudo – J20 | 11 | 21.45455 | 21.00000 | 15.00000 | 32.50000 | 5.130568 | 1.546924 |
|  | WBV – Wild Type | 10 | 21.70000 | 21.50000 | 12.00000 | 35.00000 | 6.532823 | 2.065860 |
|  | Pseudo – Wild Type | 12 | 25.00000 | 22.50000 | 18.50000 | 43.00000 | 6.960930 | 2.009447 |

| **Variable** | **Interaction** | **F value** | **P value** |
| --- | --- | --- | --- |
| **Body Weight**  **AUC** | Control / WBV | F(1.46) = 4.43 | 0.04 |
|  | J20 / WT | F(1.46) = 17.47 | 0.00 |
|  | Control / WBV / J20 / WT | F(1.46) = 0.61 | 0.43 |
| **Body Weight** | Control / WBV | F(1.46) = 3.97 | 0.052 |
|  | J20 / WT | F(1.46) = 15.06 | 0.00 |
|  | Control / WBV / J20 / WT | F(1.46) = 0.54 | 0.46 |
|  | T1 | F(1.46) = 4.87 | 0.00 |
|  | T1 * Control / WBV | F(1.46) = 0.94 | 0.45 |
|  | T1 * J20 / WT | F(1.46) = 2.78 | 0.01 |
|  | T1 * Control / WBV / J20 / WT | F(1.46) = 0.51 | 0.76 |
| **Balance**  **Beam** | Control / WBV | F(1.41) = 2.79 | 0.10 |
|  | J20 / WT | F(1.41) = 9.41 | 0.00 |
|  | Control / WBV / J20 / WT | F(1.41) = 0.16 | 0.68 |
| **GFAP**  **CA1** | Control / WBV | F(1.41) = 1.87 | 0.17 |
|  | J20 / WT | F(1.41) = 10.48 | 0.00 |
|  | Control / WBV / J20 / WT | F(1.41) = 3.05 | 0.08 |
| **GFAP**  **CA3** | Control / WBV | F(1.39) = 0.87 | 0.35 |
|  | J20 / WT | F(1.39) = 2.03 | 0.16 |
|  | Control / WBV / J20 / WT | F(1.39) = 10.58 | 0.00 |
| **GFAP**  **DG** | Control / WBV | F(1.41) = 0.84 | 0.36 |
|  | J20 / WT | F(1.41) = 2.75 | 0.10 |
|  | Control / WBV / J20 / WT | F(1.41) = 0.52 | 0.47 |
| **GFAP**  **Hilus** | Control / WBV | F(1.41) = 20.96 | 0.000 |
|  | J20 / WT | F(1.41) = 6.139 | 0.017 |
|  | Control / WBV / J20 / WT | F(1.41) = 0.323 | 0.572 |
| **CD68**  **CA1** | Control / WBV | F(1.43) = 0.720 | 0.400 |
|  | J20 / WT | F(1.43) = 6.767 | 0.012 |
|  | Control / WBV / J20 / WT | F(1.43) = 3.473 | 0.069 |
| **CD68**  **CA3** | Control / WBV | F(1.42) = 0.086 | 0.770 |
|  | J20 / WT | F(1.42) = 0.199 | 0.657 |
|  | Control / WBV / J20 / WT | F(1.42) = 0.622 | 0.434 |
| **CD68**  **DG** | Control / WBV | F(1.42) = 0.664 | 0.419 |
|  | J20 / WT | F(1.42) = 0.322 | 0.573 |
|  | Control / WBV / J20 / WT | F(1.42) = 6.999 | 0.011 |
| **CD68**  **Hilus** | Control / WBV | F(1.43) = 0.069 | 0.793 |
|  | J20 / WT | F(1.43) = 0.701 | 0.406 |
|  | Control / WBV / J20 / WT | F(1.43) = 0.121 | 0.729 |
| **IBA1**  **Activation**  **CA1** | Control / WBV | F(1.40) = 1.133 | 0.293 |
|  | J20 / WT | F(1.40) = 4.833 | 0.033 |
|  | Control / WBV / J20 / WT | F(1.40) = 1.760 | 0.192 |
| **IBA1**  **Activation**  **CA3** | Control / WBV | F(1.40) = 0.186 | 0.668 |
|  | J20 / WT | F(1.40) = 4.069 | 0.050 |
|  | Control / WBV / J20 / WT | F(1.40) = 0.487 | 0.489 |
| **IBA1**  **Activation**  **DG** | Control / WBV | F(1.40) = 5.738 | 0.021 |
|  | J20 / WT | F(1.40) = 5.940 | 0.019 |
|  | Control / WBV / J20 / WT | F(1.40) = 0.100 | 0.752 |
| **IBA1**  **Activation**  **Hilus** | Control / WBV | F(1.40) = 1.255 | 0.269 |
|  | J20 / WT | F(1.40) = 14.51 | 0.000 |
|  | Control / WBV / J20 / WT | F(1.40) = 0.493 | 0.486 |
| **IBA1**  **Cell Body**  **CA1** | Control / WBV | F(1.40) = 0.058 | 0.809 |
|  | J20 / WT | F(1.40) = 10.28 | 0.002 |
|  | Control / WBV / J20 / WT | F(1.40) = 0.148 | 0.702 |
| **IBA1**  **Cell Body**  **CA3** | Control / WBV | F(1.40) = 0.239 | 0.627 |
|  | J20 / WT | F(1.40) = 9.600 | 0.003 |
|  | Control / WBV / J20 / WT | F(1.40) = 0.046 | 0.831 |
| **IBA1**  **Cell Body**  **DG** | Control / WBV | F(1.40) = 0.000 | 0.996 |
|  | J20 / WT | F(1.40) = 9.294 | 0.004 |
|  | Control / WBV / J20 / WT | F(1.40) = 0.007 | 0.932 |
| **IBA1**  **Cell Body**  **Hilus** | Control / WBV | F(1.40) = 0.185 | 0.668 |
|  | J20 / WT | F(1.40) = 11.91 | 0.001 |
|  | Control / WBV / J20 / WT | F(1.40) = 0.501 | 0.482 |
| **IBA1**  **Dendrites**  **CA1** | Control / WBV | F(1.40) = 5.636 | 0.022 |
|  | J20 / WT | F(1.40) = 2.811 | 0.101 |
|  | Control / WBV / J20 / WT | F(1.40) = 2.030 | 0.161 |
| **IBA1**  **Dendrites**  **CA3** | Control / WBV | F(1.40) = 0.922 | 0.342 |
|  | J20 / WT | F(1.40) = 0.245 | 0.622 |
|  | Control / WBV / J20 / WT | F(1.40) = 1.628 | 0.209 |
| **IBA1**  **Dendrites**  **DG** | Control / WBV | F(1.40) = 15.15 | 0.000 |
|  | J20 / WT | F(1.40) = 0.089 | 0.766 |
|  | Control / WBV / J20 / WT | F(1.40) = 0.119 | 0.730 |
| **IBA1**  **Dendrites**  **Hilus** | Control / WBV | F(1.40) = 0.831 | 0.367 |
|  | J20 / WT | F(1.40) = 3.982 | 0.052 |
|  | Control / WBV / J20 / WT | F(1.40) = 0.024 | 0.876 |
| **IBA1**  **Coverage**  **CA1** | Control / WBV | F(1.40) = 1.015 | 0.319 |
|  | J20 / WT | F(1.40) = 6.726 | 0.013 |
|  | Control / WBV / J20 / WT | F(1.40) = 1.594 | 0.214 |
| **IBA1**  **Coverage**  **CA3** | Control / WBV | F(1.40) = 0.915 | 0.344 |
|  | J20 / WT | F(1.40) = 4.462 | 0.040 |
|  | Control / WBV / J20 / WT | F(1.40) = 1.501 | 0.227 |
| **IBA1**  **Coverage**  **DG** | Control / WBV | F(1.40) = 1.196 | 0.280 |
|  | J20 / WT | F(1.40) = 8.370 | 0.006 |
|  | Control / WBV / J20 / WT | F(1.40) = 1.617 | 0.210 |
| **IBA1**  **Coverage**  **Hilus** | Control / WBV | F(1.40) = 0.000 | 0.987 |
|  | J20 / WT | F(1.40) = 13.14 | 0.000 |
|  | Control / WBV / J20 / WT | F(1.40) = 0.421 | 0.520 |
| **IBA1**  **Cell Size**  **CA1** | Control / WBV | F(1.40) = 4.194 | 0.471 |
|  | J20 / WT | F(1.40) = 2.779 | 0.103 |
|  | Control / WBV / J20 / WT | F(1.40) = 1.176 | 0.284 |
| **IBA1**  **Cell Size**  **CA3** | Control / WBV | F(1.40) = 0.837 | 0.365 |
|  | J20 / WT | F(1.40) = 0.674 | 0.416 |
|  | Control / WBV / J20 / WT | F(1.40) = 1.494 | 0.228 |
| **IBA1**  **Cell Size**  **DG** | Control / WBV | F(1.40) = 7.447 | 0.009 |
|  | J20 / WT | F(1.40) = 0.451 | 0.505 |
|  | Control / WBV / J20 / WT | F(1.40) = 2.706 | 0.107 |
| **IBA1**  **Cell Size**  **Hilus** | Control / WBV | F(1.40) = 1.138 | 0.292 |
|  | J20 / WT | F(1.40) = 2.085 | 0.156 |
|  | Control / WBV / J20 / WT | F(1.40) = 0.137 | 0.907 |
| **IBA1**  **Cell Size**  **CA1** | Control / WBV | F(1.40) = 0.703 | 0.406 |
|  | J20 / WT | F(1.40) = 0.409 | 0.525 |
|  | Control / WBV / J20 / WT | F(1.40) = 0.144 | 0.705 |
| **IBA1**  **Cell Size**  **CA3** | Control / WBV | F(1.40) = 0.004 | 0.946 |
|  | J20 / WT | F(1.40) = 0.364 | 0.549 |
|  | Control / WBV / J20 / WT | F(1.40) = 0.127 | 0.722 |
| **IBA1**  **Cell Size**  **DG** | Control / WBV | F(1.40) = 0.208 | 0.650 |
|  | J20 / WT | F(1.40) = 1.730 | 0.195 |
|  | Control / WBV / J20 / WT | F(1.40) = 0.005 | 0.943 |
| **IBA1**  **Cell Size**  **Hilus** | Control / WBV | F(1.40) = 0.186 | 0.668 |
|  | J20 / WT | F(1.40) = 0.298 | 0.588 |
|  | Control / WBV / J20 / WT | F(1.40) = 1.234 | 0.273 |

**Body Weight**

**Figure S1.** Effects of genotype x time course (**panel A**) and time course (**panel B**) were observed on body weight. Body weight was significantly decreased from week 1 - 2 to week 3 – 6 in the wild type animals. In contrast, this effect was not observed in the J20 animals. Significant decrease of body weight (effect of tiem course) was also revealed on week 1 vs. week 3, 5 and 6.

**IBA1 Cell Body Size**

**IBA1 Dendrites Size**

**IBA1 Total Coverage**
